# Surgical Management of Primary Rhegmatogenous Retinal Detachment: Experiences from a Serbian Tertiary Center

**DOI:** 10.3390/life16101617

**Published:** 2026-09-26

**Authors:** Vladimir Milutinovic, Dragan Spaic, Jelena Kostic, Aleksandar Risimic, Marko Svetel, Bozidar Savic, Borivoje Savic

**Affiliations:** 1 Clinic for Eye Diseases, University Clinical Center of Serbia, 11000 Belgrade, Serbia; dr.vladimir.milutinovic@gmail.com (V.M.);; 2 Faculty of Medicine Foča, University of East Sarajevo, 73300 Foča, Bosnia and Herzegovina; 3 Institute of Veterinary Medicine of Serbia, Janisa Janulisa 14, 11000 Belgrade, Serbia

**Keywords:** rhegmatogenous retinal detachment, pars plana vitrectomy, scleral buckling, pneumatic retinopexy, best-corrected visual acuity, retinal re-detachment, vitreoretinal surgery

## Abstract

(1) Background: Rhegmatogenous retinal detachment (RRD) requires timely surgical repair, most commonly using pars plana vitrectomy (PPV), scleral buckling (SB), or pneumatic retinopexy (PR). Surgical practice varies across regions, while contemporary real-world data from Southeastern Europe remain limited. This study evaluated contemporary surgical management and 12-month outcomes of primary RRD at a Serbian tertiary referral center. (2) Methods: This retrospective, single-center observational study included 188 patients who underwent primary RRD repair between January and December 2024. Patients were treated with PPV, SB, or PR and followed for up to 12 months. Best-corrected visual acuity (BCVA) was assessed preoperatively and at postoperative day 1, day 7, and at 1, 3, 6, and 12 months. Longitudinal BCVA changes were analyzed using a linear mixed-effects model, with generalized estimating equations used as a complementary population-averaged analysis. Retinal re-detachment and postoperative intraocular pressure (IOP) elevation were also evaluated. (3) Results: PPV was performed in 104 patients (55.3%), SB in 48 (25.5%), and PR in 36 (19.1%). Baseline characteristics differed significantly among groups, including age, lens status, macular status, symptom duration, and preoperative BCVA. Longitudinal analysis demonstrated significant effects of time (F = 119.53, *p* < 0.001), macular status (F = 86.36, *p* < 0.001), symptom duration (F = 2.93, *p* = 0.035), and a significant time-by-surgery interaction (F = 4.86, *p* = 0.009). Model-estimated BCVA at 12 months was 0.77 logMAR after PPV, 0.58 after SB, and 0.28 after PR. However, PR patients had more favorable baseline characteristics. Retinal re-detachment occurred in 18.6% of patients, with no significant difference among surgical groups (*p* = 0.246). Early postoperative IOP elevation was significantly more frequent after PPV (22.3%) than after SB (0%) or PR (2.9%) (*p* < 0.001). (4) Conclusions: All three surgical approaches were associated with substantial visual recovery during 12 months of follow-up. PPV was the predominant surgical strategy, while SB remained frequently used and PR provided favorable outcomes in carefully selected patients. Because of substantial baseline differences among treatment groups, between-group outcome differences should not be interpreted as causal evidence of surgical superiority. Macular status and symptom duration were important determinants of functional recovery.

## 1. Introduction

Rhegmatogenous retinal detachment (RRD) is a vision-threatening vitreoretinal disorder that requires timely surgical intervention to restore retinal anatomy and minimize irreversible visual impairment. The principal surgical approaches include scleral buckling (SB), pars plana vitrectomy (PPV), and pneumatic retinopexy (PR). Although all three techniques can achieve high rates of primary retinal reattachment when appropriately selected, their utilization varies considerably according to patient characteristics, retinal anatomy, surgeon experience, and regional practice patterns [1,2,3].

Scleral buckling was established as the standard surgical treatment for RRD following its introduction in the 1950s. However, the development and subsequent refinement of pars plana vitrectomy led to a progressive change in surgical practice. Historical data demonstrate that PPV accounted for only approximately 1% of RRD procedures in 1979–1980 but increased to 63% by 1999 [4]. This transition continued over subsequent decades. In the United States, PPV accounted for approximately 83% of RRD procedures by 2014, whereas SB and PR accounted for approximately 5% and 12%, respectively [5]. A similar shift toward vitrectomy-based repair has been reported in large tertiary-center and national datasets from Singapore, Spain, and the United Kingdom [6,7,8].

Pneumatic retinopexy, introduced in the mid-1980s, represents a less invasive alternative for selected RRD configurations. Although its use remains less frequent than that of PPV, PR can provide favorable anatomical and functional outcomes in appropriately selected patients. Its broader application is limited primarily by specific anatomical eligibility criteria, the need for careful patient selection and postoperative positioning, and the possibility of additional intervention following primary failure [9].

Despite the global predominance of PPV, substantial regional variation in RRD management persists. Previous data from Serbia demonstrated that SB remained the more frequently used surgical approach in 2016, accounting for 57% of primary RRD procedures compared with 43% for PPV [10]. These findings suggested a slower transition from conventional extraocular surgery toward vitrectomy-based management than that observed in many other healthcare systems. Contemporary data describing whether this surgical pattern has subsequently changed in Serbia are limited.

Selection of the optimal surgical approach for an individual patient remains multifactorial. Important considerations include age, lens status, macular involvement, the number and location of retinal breaks, the extent and configuration of retinal detachment, symptom duration, vitreoretinal characteristics, surgeon experience, and the availability of surgical resources [11]. Consequently, direct comparisons of outcomes among SB, PPV, and PR in observational cohorts are inherently influenced by differences in baseline clinical characteristics and by treatment-selection bias. Longitudinal assessment of postoperative visual recovery therefore requires statistical approaches capable of accounting, at least in part, for repeated measurements and clinically relevant baseline differences between treatment groups.

Although numerous studies have evaluated the surgical management of RRD, contemporary real-world evidence from Southeastern Europe remains limited, particularly regarding changes in surgical practice and longitudinal functional outcomes following different primary repair strategies. Evaluating such data may provide insight not only into anatomical and visual outcomes but also into evolving regional surgical patterns and the continued role of SB and PR in the era of increasingly predominant vitrectomy.

Therefore, the present study aimed to characterize contemporary real-world surgical management of primary RRD at a tertiary referral center in Serbia, including the utilization of PPV, SB, and PR and the clinical profiles of patients selected for each approach, and to describe the longitudinal functional and anatomical outcomes observed during 12 months of follow-up. Between-group analyses were intended to characterize differences in patient profiles and observed outcome patterns among clinically selected surgical groups and were not designed to estimate causal treatment effects or comparative surgical efficacy. An additional aim was to assess contemporary procedural utilization in the context of previously reported Serbian practice patterns. The primary outcome was best-corrected visual acuity (BCVA) at 12 months after surgery. Secondary outcomes included the longitudinal pattern of visual recovery, retinal re-detachment, and postoperative complications, with particular emphasis on early postoperative intraocular pressure elevation.

## 2. Materials and Methods

### 2.1. Study Design and Setting

This retrospective, single-center observational study included patients who underwent primary surgical treatment for rhegmatogenous retinal detachment (RRD) at the Clinic for Eye Diseases, University Clinical Center of Serbia, Belgrade, Serbia, between 1 January and 31 December 2024. Clinical and surgical data were retrospectively obtained from the institutional electronic medical record system. Postoperative outcomes were assessed during a follow-up period of up to 12 months.

All surgical procedures were performed by vitreoretinal surgeons with at least five years of experience in vitreoretinal surgery. A total of 188 patients met the eligibility criteria and were included in the analysis.

The study was conducted in accordance with the principles of the Declaration of Helsinki. Written informed consent was obtained from all patients for the use of their clinical data for scientific purposes. Patient confidentiality was maintained throughout data collection and analysis, and no directly identifying patient information was included in the analytical dataset.

### 2.2. Eligibility Criteria

Patients were eligible for inclusion if they had primary RRD and underwent primary surgical repair during the predefined study period.

The inclusion criteria were as follows:

- Primary rhegmatogenous retinal detachment;
- Primary surgical treatment performed between 1 January and 31 December 2024;
- Availability of sufficient clinical and postoperative follow-up data for assessment of the predefined study outcomes.

The exclusion criteria were as follows:

- Tractional or exudative retinal detachment;
- Recurrent retinal detachment at presentation;
- Previous surgical treatment for retinal detachment in the affected eye;
- Incomplete medical records that precluded reliable assessment of the predefined study variables;
- Insufficient postoperative follow-up data for outcome assessment.

### 2.3. Data Collection and Clinical Variables

Demographic, ophthalmological, surgical, and postoperative data were extracted from the medical records using a predefined set of variables. The recorded demographic and baseline clinical characteristics included age, sex, affected eye, lens status, macular status at presentation, duration of symptoms before surgery, and preoperative best-corrected visual acuity (BCVA).

Lens status was categorized as phakic or pseudophakic. Previous neodymium-doped yttrium aluminum garnet (Nd) laser posterior capsulotomy was additionally recorded in pseudophakic eyes.

Macular status at presentation was categorized as macula-on or macula-off according to whether the macula remained attached or was involved by the retinal detachment.

Duration of symptoms was defined as the interval, expressed in days, between the patient-reported onset of visual symptoms attributable to retinal detachment and primary surgical treatment.

BCVA was measured using Snellen visual acuity charts during routine ophthalmological examinations and converted to logarithm of the minimum angle of resolution (logMAR) units for statistical analysis. BCVA measurements were recorded preoperatively and on postoperative day 1, postoperative day 7, and at 1, 3, 6, and 12 months after surgery.

Postoperative data included BCVA at each available follow-up visit, intraocular pressure (IOP), retinal re-detachment, and other documented postoperative complications.

### 2.4. Surgical Procedures

The surgical technique was not randomly assigned. The choice of procedure was made by the operating vitreoretinal surgeon on the basis of the individual clinical characteristics of the retinal detachment, including lens status, macular involvement, retinal break characteristics, retinal detachment configuration, and overall clinical assessment.

PPV was performed using a 23-gauge transconjunctival vitreoretinal system (ACCURUS^®^ Surgical System, Alcon Laboratories, Inc., Fort Worth, TX, USA). Standard 23-gauge vitreoretinal instruments and consumables were used according to the surgical requirements of each case. Depending on the clinical indication, PPV was performed either alone or in combination with phacoemulsification and intraocular lens implantation. Intraocular tamponade consisted of either silicone oil or gas, including sulfur hexafluoride (SF6) or perfluoropropane (C3F8), according to the intraoperative findings and the surgeon’s clinical judgment. Commercial vitreoretinal tamponade products were obtained through the institutional procurement system. Because this was a retrospective study, the specific commercial manufacturer and lot number of the tamponade agent used in each individual procedure were not consistently recorded in the electronic medical records.

In SB procedures, a 2-mm silicone encircling band and a 7-mm silicone explant were used according to the location and characteristics of the retinal pathology. These commercially available ophthalmic silicone implants were supplied through the institutional procurement system; however, the specific manufacturer and lot number used in each individual case were not consistently documented in the retrospective electronic medical records.

PR was performed in selected patients considered anatomically suitable for this approach on the basis of retinal detachment configuration and retinal break characteristics.

For analyses of observed outcome patterns according to primary surgical strategy, patients were classified into three clinically selected groups: PPV, SB, and PR. The PPV group included PPV performed with or without concomitant phacoemulsification and with either silicone oil or gas tamponade.

### 2.5. Outcome Measures

The primary outcome was BCVA, expressed in logMAR units, at 12 months after primary RRD repair.

Secondary outcomes included the longitudinal trajectory of BCVA during the 12-month postoperative period, retinal re-detachment following primary surgical repair, and postoperative complications, with particular emphasis on early postoperative IOP elevation.

Elevated postoperative IOP was defined as an IOP > 21 mmHg. Its incidence was evaluated according to the primary surgical procedure. Within the PPV group, the occurrence of IOP elevation was additionally compared between eyes receiving silicone oil and those receiving gas tamponade.

Retinal re-detachment was defined as recurrent separation of the neurosensory retina after primary surgical repair documented during the postoperative follow-up period. Re-detachment occurring during the 12-month follow-up period was recorded as an anatomical failure of the primary repair.

### 2.6. Statistical Analysis

Categorical variables are presented as absolute frequencies and percentages. Continuous variables are presented as medians and interquartile ranges (IQRs). The distribution of continuous variables was assessed using the Kolmogorov–Smirnov test, and non-parametric methods were used for variables showing non-normal distributions.

For comparisons between two independent groups, continuous variables were analyzed using the Mann–Whitney U test. Comparisons of continuous variables among the three surgical groups were performed using the Kruskal–Wallis test. When a statistically significant overall difference was detected, pairwise post hoc comparisons were performed with Bonferroni adjustment for multiple comparisons.

Categorical variables were compared using the chi-square test. Where appropriate, Cramér’s V was calculated to quantify the strength of association between categorical variables.

Because BCVA was repeatedly measured in the same patients during follow-up, longitudinal visual outcomes were primarily evaluated using a linear mixed-effects model (LMM). BCVA expressed in logMAR units was entered as the dependent variable. Time, surgical technique, macular status, and duration of symptoms were included as fixed effects. The interaction between time and surgical strategy was included to explore whether the observed pattern of visual recovery over time differed among the clinically selected PPV, SB, and PR groups.

Model-derived estimated marginal means were used to characterize changes in BCVA over time and differences in visual recovery among surgical groups. The 12-month BCVA values were used to characterize the primary functional endpoint, whereas the complete series of repeated BCVA measurements was used to evaluate the postoperative visual recovery trajectory.

As a complementary population-averaged analysis, generalized estimating equations (GEE) were applied to the longitudinal BCVA data. A linear response model with an exchangeable working correlation structure was used, with time, surgical technique, and the interaction between surgical technique and time included as model effects. The GEE analysis was used to assess the consistency of the longitudinal findings obtained from the mixed-effects analysis at the population level.

Longitudinal analyses were based on the available repeated BCVA observations; missing observations at individual follow-up visits were not included in the corresponding analyses.

Because the surgical procedure was selected according to clinical indications rather than by random allocation, the three surgical groups represent clinically distinct patient populations. Accordingly, between-group analyses were used to characterize differences in observed longitudinal outcome patterns among patients managed with different clinically selected surgical strategies and were not intended to estimate causal treatment effects or comparative surgical efficacy. Although macular status and symptom duration were included as clinically relevant covariates in the longitudinal model, residual confounding by indication and by other baseline characteristics cannot be excluded. These covariates were included as clinically relevant determinants of postoperative visual recovery; the model was intended to characterize longitudinal outcome trajectories rather than to provide a fully adjusted estimate of causal comparative treatment effectiveness.

Retinal re-detachment and postoperative IOP elevation were analyzed as secondary categorical outcomes. Re-detachment rates were compared among the PPV, SB, and PR groups using the chi-square test. The incidence of postoperative IOP elevation was compared among surgical groups and, within the PPV group, according to intraocular tamponade type.

All statistical tests were two-sided, and a *p*-value < 0.05 was considered statistically significant. Statistical analyses were performed using IBM SPSS Statistics, version 29.0 (IBM Corp., Armonk, NY, USA).

## 3. Results

### 3.1. Patient Characteristics

A total of 188 patients with primary rhegmatogenous retinal detachment (RRD) were included in the analysis. Of these, 124 (66.0%) were male and 64 (34.0%) were female. The median age was 60 years (IQR, 17) in male patients and 64 years (IQR, 14) in female patients, with no statistically significant difference between sexes (U = 3305, Z = −1.876, *p* = 0.061).

The right eye was affected in 89 patients (47.3%) and the left eye in 99 patients (52.7%). Most eyes were phakic (*n* = 139, 73.9%), whereas 49 eyes (26.1%) were pseudophakic. Four pseudophakic eyes (2.1% of the total cohort) had previously undergone Nd laser posterior capsulotomy.

At presentation, the macula was detached in 132 patients (70.2%) and remained attached in 56 patients (29.8%). Amblyopia was present in 6 patients (3.2%), and glaucoma was documented in 8 patients (4.3%).

The median preoperative BCVA was 1.78 logMAR (IQR, 2.479), and the median duration of symptoms before surgical treatment was 7 days (IQR, 12).

### 3.2. Distribution of Surgical Procedures

PPV was the most frequently performed primary surgical procedure and was used in 104 patients (55.3%). SB was performed in 48 patients (25.5%), and PR in 36 patients (19.1%).

Among patients undergoing PPV, 82 received silicone oil tamponade and 22 received gas tamponade. Combined phacoemulsification and PPV was performed in 48 patients.

The detailed distribution of surgical procedures is presented in Table 1.

### 3.3. Baseline Characteristics According to Surgical Procedure

The three surgical groups differed significantly in several baseline clinical characteristics. No statistically significant association was observed between surgical procedure and sex (*p* = 0.553), and no relevant difference was observed with respect to eye laterality.

Age differed significantly among the surgical groups (*p* = 0.003). Patients undergoing PR were younger, with a median age of 57.5 years (IQR, 15), compared with 62.0 years (IQR, 14) in the SB group and 64.5 years (IQR, 15) in the PPV group.

A strong association was observed between lens status and surgical technique (*p* < 0.001). Among pseudophakic eyes, 85.7% were treated with PPV, compared with 8.2% with SB and 6.1% with PR. Among phakic eyes, PPV accounted for 44.6% of procedures, SB for 31.7%, and PR for 23.7%.

Macular status was also significantly associated with surgical technique (*p* < 0.001). Among patients with macula-off RRD, 65.9% underwent PPV, 23.5% underwent SB, and 10.6% underwent PR. Conversely, among patients with macula-on RRD, PR was the most frequently used procedure (39.3%), whereas PPV and SB were each performed in 30.4% of cases.

The median duration of symptoms differed significantly among groups (*p* = 0.018), with values of 7 days (IQR, 18) for PPV, 9 days (IQR, 10) for SB, and 4 days (IQR, 8) for PR. Post hoc analysis showed a significantly shorter duration of symptoms in the PR group than in the SB group (*p* = 0.019).

Preoperative BCVA also differed significantly among the three groups (*p* < 0.001). Median preoperative BCVA was 2.08 logMAR (IQR, 2.02) in the PPV group, 1.48 logMAR (IQR, 2.32) in the SB group, and 0.301 logMAR (IQR, 1.66) in the PR group. Post hoc comparisons showed significantly better preoperative BCVA in the PR group than in the SB group (*p* = 0.046) and the PPV group (*p* < 0.001).

Baseline characteristics according to surgical technique are summarized in Table 2.

### 3.4. Longitudinal Visual Outcomes: Linear Mixed-Effects Model

Longitudinal changes in BCVA were first evaluated using the linear mixed-effects model.

A significant effect of time on BCVA was observed (F = 119.53, *p* < 0.001), demonstrating substantial visual recovery during postoperative follow-up. Macular status was strongly associated with BCVA (F = 86.36, *p* < 0.001), and symptom duration also showed a statistically significant effect (F = 2.93, *p* = 0.035).

Importantly, a significant interaction between time and surgical technique was observed (F = 4.86, *p* = 0.009), indicating that the pattern of visual recovery over time differed among the three surgical groups. This finding describes differences in observed longitudinal recovery patterns among clinically selected treatment groups and should not be interpreted as evidence of a causal effect of surgical technique.

During the early postoperative period, model-estimated mean BCVA improved from 1.275 logMAR preoperatively to 0.497 logMAR at 1 month (*p* < 0.001).

Pairwise analysis of early postoperative recovery demonstrated a difference of 0.298 logMAR between PR and PPV in favor of PR (*p* = 0.019). Macula-off status was associated with BCVA that was poorer by 0.758 logMAR (*p* < 0.001).

During subsequent follow-up, the magnitude of visual improvement became smaller, whereas macular status remained strongly associated with visual outcome, with a mean difference of 0.440 logMAR (*p* < 0.001).

These findings indicate that postoperative BCVA improved substantially over time and that the observed trajectory of recovery differed according to surgical strategy, baseline macular status, and symptom duration.

### 3.5. Complementary Longitudinal Analysis Using Generalized Estimating Equations

The complementary GEE analysis across all available follow-up time points was consistent with the presence of significant longitudinal changes in BCVA.

Significant effects were observed for time (χ^2^ = 213.76, *p* < 0.001), surgical technique (χ^2^ = 36.96, *p* < 0.001), and the interaction between surgical technique and time (χ^2^ = 22.82, *p* = 0.029).

Across the follow-up period, the model-estimated mean BCVA was 0.496 ± 0.08 logMAR in the PR group, 0.864 ± 0.08 logMAR in the SB group, and 1.068 ± 0.05 logMAR in the PPV group. Pairwise comparisons demonstrated lower logMAR values in the PR group than in the SB group (*p* = 0.005) and the PPV group (*p* < 0.001).

Because the three treatment groups differed substantially in baseline disease characteristics and the surgical procedure was not randomly assigned, these between-group differences represent associations with surgical strategy and should not be interpreted as causal treatment effects.

The estimated BCVA values at individual follow-up time points are presented in Table 3.

At 12 months, the model-estimated BCVA was 0.77 logMAR in the PPV group, 0.58 logMAR in the SB group, and 0.28 logMAR in the PR group. These final visual outcomes should be interpreted together with the substantial differences in preoperative BCVA, macular status, lens status, age, and duration of symptoms among the treatment groups.

### 3.6. Postoperative Intraocular Pressure Elevation

Early postoperative IOP elevation differed significantly among surgical groups.

At postoperative day 7, IOP > 21 mmHg was documented in 22.3% of patients undergoing PPV, compared with 0% of patients undergoing SB and 2.9% of patients undergoing PR (χ^2^ = 18.399, *p* < 0.001). The strength of the association between surgical procedure and postoperative IOP elevation was quantified by a Cramér’s V of 0.315.

Within the PPV group, no statistically significant difference in postoperative IOP elevation was detected between eyes receiving silicone oil and those receiving gas tamponade (*p* > 0.05).

These findings are summarized in Table 4.

### 3.7. Retinal Re-Detachment

Retinal re-detachment during follow-up occurred in 35 of 188 patients (18.6%), corresponding to an overall single-surgery anatomical attachment rate of 81.4%.

The reported re-detachment rate refers to events documented during the predefined 12-month postoperative follow-up period.

The re-detachment rate was 14.4% in the PPV group, 25.0% in the SB group, and 22.2% in the PR group (Table 5). Although the numerical rate was lowest following PPV, the difference among the three surgical groups was not statistically significant (χ^2^ = 2.807, *p* = 0.246).

Accordingly, no statistically significant association between the primary surgical technique and retinal re-detachment was demonstrated in this cohort.

## 4. Discussion

This study provides contemporary real-world data on the surgical management of primary rhegmatogenous retinal detachment (RRD) at a tertiary referral center in Serbia. PPV was the most frequently used procedure, accounting for 55.3% of cases, followed by SB in 25.5% and PR in 19.1%. Visual acuity improved substantially during follow-up in all three surgical groups. However, the groups differed considerably in baseline characteristics, particularly macular status, lens status, preoperative BCVA, age, and symptom duration, emphasizing the importance of cautious interpretation of direct between-group comparisons.

Patients treated with PR demonstrated the best model-estimated visual acuity during follow-up and at 12 months. Nevertheless, these patients also had substantially more favorable baseline characteristics, including better preoperative BCVA, shorter symptom duration, and a higher proportion of macula-on detachments. Therefore, the observed visual advantage associated with PR should not be interpreted as evidence of superiority over PPV or SB. Rather, it reflects both appropriate patient selection and differences in baseline disease severity. This interpretation is consistent with the PIVOT trial and subsequent systematic analyses showing favorable functional outcomes with PR in carefully selected patients [12,13,14].

Macular status was one of the strongest factors associated with postoperative visual outcome in our cohort. Macula-off RRD was associated with significantly poorer BCVA, while a longer duration of symptoms was also related to less favorable recovery. These findings are consistent with previous studies demonstrating that foveal involvement and timing of surgical repair are major determinants of functional prognosis following RRD surgery [15,16]. The significant interaction between surgical strategy and time observed in the longitudinal model suggests that the observed pattern of visual recovery differed among the clinically selected treatment groups. However, because treatment allocation was based on clinical indications and baseline characteristics differed substantially among groups, this finding should be interpreted as an observational difference in recovery patterns rather than as evidence that the surgical procedure itself caused the difference.

The overall retinal re-detachment rate was 18.6%, corresponding to a single-surgery anatomical attachment rate of 81.4%. Although re-detachment was numerically less frequent after PPV than after SB or PR, the difference was not statistically significant. These findings are generally consistent with previous studies showing high anatomical success rates with all established RRD repair techniques when appropriately selected [17,18,19,20]. Lens status strongly influenced surgical selection in our cohort, with PPV used in 85.7% of pseudophakic eyes, whereas SB was predominantly performed in phakic eyes. This pattern is consistent with the established role of PPV in pseudophakic RRD and the continued value of SB in selected younger and phakic patients in whom preservation of the crystalline lens is desirable [18,19,20,21].

A clinically relevant finding was the significantly higher incidence of early postoperative IOP elevation following PPV. At postoperative day 7, IOP > 21 mmHg occurred in 22.3% of PPV-treated eyes compared with 0% after SB and 2.9% after PR. Although no significant difference was detected between silicone oil and gas tamponade within the PPV group, these findings support careful IOP monitoring during the early postoperative period after vitrectomy. The absence of early IOP elevation in the SB group may also be relevant when selecting among otherwise appropriate surgical options in patients considered vulnerable to postoperative ocular hypertension.

An additional finding of interest is the continued substantial use of SB in Serbia. Previous Serbian data reported SB use in approximately 57% of RRD procedures in 2016 [10], whereas the present cohort showed a reduction to 25.5% in 2024. This suggests a clear shift toward vitrectomy-based management while demonstrating that SB remains an important component of contemporary vitreoretinal practice at our institution. Preservation of expertise in SB may be particularly valuable for selected phakic patients and for maintaining a broader range of surgical options in RRD management.

This study has several limitations. Its retrospective and single-center design introduces the possibility of selection bias and limits generalizability. Surgical treatment was not randomly assigned, and important baseline differences existed among the three treatment groups, resulting in unavoidable confounding by indication. Although the longitudinal model incorporated macular status and symptom duration, residual confounding related to other baseline characteristics, including age, lens status, preoperative BCVA, and retinal anatomical features, cannot be excluded. In addition, the PPV group was heterogeneous with respect to concomitant phacoemulsification and tamponade type. Accordingly, the practical value of the present study lies not in proposing changes to current RRD treatment guidelines, but in providing contemporary real-world information on surgical selection and postoperative outcomes in a tertiary-care setting. The findings highlight the continued importance of matching the surgical approach to the clinical characteristics of the detachment, reinforce the prognostic relevance of macular status and symptom duration, and support careful early IOP monitoring after PPV. Nevertheless, the relatively large real-world cohort, 12-month follow-up, repeated longitudinal assessment, and inclusion of all three commonly used primary surgical strategies provide a clinically relevant characterization of contemporary RRD management at a Serbian tertiary referral center. The principal value of the study lies in describing current procedural utilization, patient selection patterns, longitudinal outcomes, and changes in regional surgical practice rather than in estimating comparative treatment effectiveness.

## 5. Conclusions

In this contemporary single-center cohort, PPV was the most frequently used surgical approach for primary rhegmatogenous retinal detachment, while SB remained an important treatment option and PR was used in selected patients with more favorable baseline characteristics. Visual acuity improved substantially during the 12-month follow-up period, with macular status and symptom duration representing important determinants of functional recovery.

Although PR was associated with the best model-estimated visual outcomes, substantial baseline differences among treatment groups preclude causal conclusions regarding the superiority of any individual surgical technique. Retinal re-detachment rates did not differ significantly among PPV, SB, and PR, whereas early postoperative IOP elevation was more frequent following PPV.

These findings characterize contemporary real-world surgical selection and longitudinal outcomes in primary RRD at a Serbian tertiary referral center. They also demonstrate an ongoing shift toward vitrectomy-based management compared with previously reported Serbian practice patterns, while confirming the continued clinical role of scleral buckling and pneumatic retinopexy in selected patients. Between-group differences should be interpreted as observational findings arising from clinically selected patient populations and not as evidence of comparative surgical superiority. The clinical relevance of these findings lies in informing patient selection, postoperative monitoring, and interpretation of contemporary real-world RRD practice rather than in supporting changes to current treatment guidelines.

## Figures and Tables

**Table 1:** Distribution of patients according to primary surgical procedure.

Surgical Procedure	*n*	%	Cumulative %
Phacoemulsification + PPV + silicone oil	40	21.3	21.3
PPV + silicone oil	42	22.3	43.6
Phacoemulsification + PPV + gas	8	4.3	47.9
PPV + gas	14	7.4	55.3
Scleral buckling	48	25.5	80.9
Pneumatic retinopexy	36	19.1	100.0

**Table 2:** Baseline demographic and clinical characteristics according to primary surgical procedure.

Variable	PPV	SB	PR	*p*-Value
Male sex, *n* (%)	70 (56.5)	33 (26.6)	21 (16.9)	0.553
Age, years, median (IQR)	64.5 (15)	62.0 (14)	57.5 (15)	0.003
Lens status				<0.001
Phakic	62 (44.6)	44 (31.7)	33 (23.7)	
Pseudophakic	42 (85.7)	4 (8.2)	3 (6.1)	
Macular status				<0.001
Macula-on	17 (30.4)	17 (30.4)	22 (39.3)	
Macula-off	87 (65.9)	31 (23.5)	14 (10.6)	
Symptom duration, days, median (IQR)	7 (18)	9 (10)	4 (8)	0.018
Preoperative BCVA, logMAR, median (IQR)	2.08 (2.02)	1.48 (2.32)	0.301 (1.66)	<0.001

Percentages for sex, lens status, and macular status are presented according to the corresponding category distribution across surgical groups. BCVA, best-corrected visual acuity; IQR, interquartile range; PPV, pars plana vitrectomy; PR, pneumatic retinopexy; SB, scleral buckling.

**Table 3:** Model-estimated BCVA (logMAR) according to surgical procedure and follow-up time.

Follow-Up	PPV	SB	PR
Preoperative	1.79 (1.60–1.98)	1.40 (1.10–1.70)	0.87 (0.55–1.20)
Day 1	1.58 (1.42–1.74)	1.24 (1.01–1.47)	0.77 (0.49–1.06)
Day 7	1.03 (0.90–1.17)	0.82 (0.63–1.00)	0.40 (0.24–0.56)
1 month	0.74 (0.64–0.84)	0.70 (0.53–0.88)	0.30 (0.16–0.45)
3 months	0.76 (0.65–0.86)	0.63 (0.47–0.80)	0.47 (0.19–0.74)
6 months	0.81 (0.68–0.93)	0.67 (0.38–0.97)	0.37 (0.15–0.59)
12 months	0.77 (0.60–0.93)	0.58 (0.44–0.72)	0.28 (0.04–0.53)

Values are presented as model-estimated means with 95% confidence intervals. Lower logMAR values indicate better visual acuity. BCVA, best-corrected visual acuity; PPV, pars plana vitrectomy; PR, pneumatic retinopexy; SB, scleral buckling.

**Table 4:** Early postoperative intraocular pressure elevation according to primary surgical procedure.

Surgical Procedure	IOP > 21 mmHg at Day 7
PPV	22.3%
SB	0%
PR	2.9%

Overall comparison: χ^2^ = 18.399, *p* < 0.001; Cramér’s V = 0.315. IOP, intraocular pressure; PPV, pars plana vitrectomy; PR, pneumatic retinopexy; SB, scleral buckling.

**Table 5:** Retinal re-detachment according to primary surgical procedure.

Surgical Procedure	Re-Detachment Rate
PPV	14.4%
SB	25.0%
PR	22.2%
Overall	18.6%

Comparison among surgical groups: χ^2^ = 2.807, *p* = 0.246. PPV, pars plana vitrectomy; PR, pneumatic retinopexy; SB, scleral buckling.

## Data Availability

The raw data supporting the conclusions of this article will be made available by the authors on request.
